# A Patient with Corneal Epithelial Disorder That Developed after Administration of a Latanoprost Generic, but Not a Brand-Name Drug, Eye Drop

**DOI:** 10.1155/2012/536746

**Published:** 2012-10-02

**Authors:** Yukihisa Takada, Yuka Okada, Norihito Fujita, Shizuya Saika

**Affiliations:** Department of Ophthalmology, School of Medicine, Wakayama Medical University, 811-1 Kimiidera, Wakayama 641-0012, Japan

## Abstract

*Background*. We report a patient who developed corneal epithelial disorder repeatedly after changing the prescription from Xalatan eye drops (Pfizer Inc.) to Latanoprost eye drops (Kaken Pharmaceutical Co., Ltd.), both containing 0.005% latanoprost. *Case Report*. An 88-year-old male with glaucoma had been treated with Timoptol eye drops and Xalatan eye drops for a few years. While he stayed in a health care facility for the elderly, Xalatan eye drops was changed to Latanoprost eye drops usage, and eye pain developed on the day of this change. On the next day, he visited our department, and corneal epithelial disorder was observed. The drops were discontinued, and the corneal epithelial disorder healed after 2 days. Twenty days after the first consultation, Xalatan eye drops and Latanoprost eye drops were resumed by a physician of internal medicine in the health care facility, but eye pain developed again. After discontinuation of the two drugs, Xalatan eye drops usage was resumed the next day, but no corneal epithelial disorder was observed thereafter. *Conclusions*. This clinical history strongly suggested the association between a generic drug, Latanoprost eye drops, and the development of corneal epithelial disorder.

## 1. Introduction

Among various antiglaucoma eye drops, prostaglandin (PG) preparations are often prescribed as the first choice due to their marked eye pressure-decreasing effects. However, as an adverse effect of PG preparations, corneal disorder has been reported, and its cause has been considered to be the product itself [1] or benzalkonium chloride contained in the product [2, 3].

Commercially available PG preparations are classified into prostone and prost types. The patent (patent no. 2721414) for Xalatan eye drops (0.005% latanoprost, Pfizer Inc.), which is a Latanoprost compound as a prost type, expired on September 6, 2009, and 22 generic drugs of 22 companies were commercially available as of May 2010. In Japan, generic and brand-name drugs are the same only in the main component and sometimes differ in preservatives or additives. However, fewer clinical tests are performed on generic than on brand-name drugs.

 We report a patient who repeatedly developed corneal disorder following a change from Xalatan eye drops as a brand-name drug to Latanoprost eye drops (Kaken Pharmaceutical Co., Ltd., Japan) as its generic drug.

## 2. Case Presentation

### 2.1. Patient

 An 88-year-old male with a past history of diabetes mellitus, hypertension, and dementia underwent cataract surgery 4-5 years previously.

A few years earlier, Xalatan eye drops usage was prescribed for the right eye and Timolol XE eye drops (0.5% timolol malate) and Xalatan eye drops for the left eye in a local hospital. After he was admitted to a health care facility for the elderly due to dementia, Xalatan eye drops usage was changed to Latanoprost eye drops (Kaken Pharmaceutical Co., Ltd.) on July 15, 2010 by a physician of internal medicine in the facility. He noticed pain in the left eye at night on the day of the change in eye drops, visited a local hospital the next day, and was diagnosed with corneal disorder and referred to our department.

### 2.2. Findings at the Initial Examination in Our Department Clinic

**Right Eye**

There were no abnormalities in the palpebral or bulbar conjunctiva. The cornea was clear, and no inflammatory cells were observed in the anterior chamber. The intraocular lens had been fixed in the capsular bag. There were no markedly abnormal findings in the fundus. The intraocular pressure was 9 mmHg.

**Left Eye**

**Figure 1:**
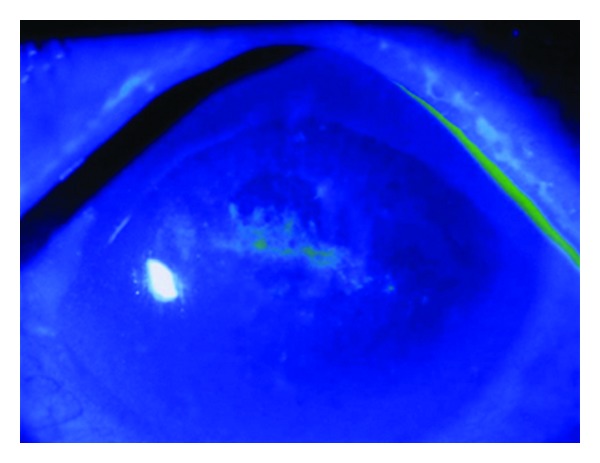
Fluorescein staining at the initial consultation. Corneal erosion and an epithelial crack line were observed in the corneal center.

There were no abnormalities in the palpebral or bulbar conjunctiva. A corneal epithelial defect and an epithelial crack line were observed in the center of the cornea (Figure 1). There were no inflammatory cells in the anterior chamber. The intraocular lens was fixed in the capsular bag. There were no markedly abnormal findings in the fundus. The intraocular pressure was 15 mmHg.

The lacrimal secretion function evaluated using the Schirmer I test was 5 mm in the right eye and 20 mm in the left eye. Esthesiometry (Cochet-Bonnet aesthesiometer, Handaya Co., Ltd., Tokyo, Japan) showed corneal sensitivity of 50 mm in the right eye and 20 mm in the left eye.

### 2.3. Clinical Course

**Figure 2:**
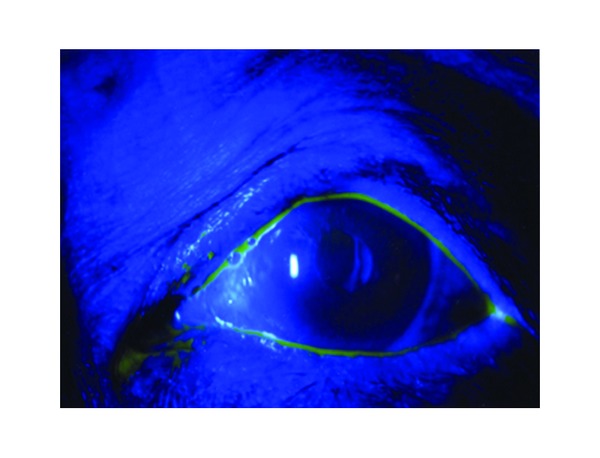
Fluorescein staining at the final consultation. Neither marked staining nor retention of fluorescein was observed.

Drug-induced corneal disorder was initially suspected. After the discontinuation of Timoptol XE eye drops and Latanoprost eye drops, Tarivid ophthalmic ointment (left eye, once daily) was prescribed. After 2 days, the corneal disorder improved, and, after 34 days, an instruction was given to resume the administration of only Timoptol XE eye drops into the left eye. However, in the health care facility for the elderly where he stayed, the administration of Timoptol XE eye drops (left eye, once daily) and Latanoprost eye drops (left eye, once daily) was resumed by mistake, and pain in the left eye developed. Three days after resumption of the eye drops, only Latanoprost eye drops usage was discontinued by a physician of internal medicine in the facility, and the eye pain disappeared. He visited our department 5 days after the discontinuation of the eye drops, and no corneal disorder was observed. Therefore, an instruction to resume Xalatan eye drops was given. Subsequent observation of the course revealed no corneal disorder (Figure 2).

## 3. Discussion

We encountered a patient who developed corneal disorder after a change from Xalatan eye drops to Latanoprost eye drops as a generic drug. Previous studies have shown corneal disorder due to PG preparations, decreased sensitivity or aggravation of the lacrimal fluid environment due to *β*-blockers [4], and aggravation of corneal disorder due to combination therapy with PG preparations and *β*-blockers [5]. In addition, corneal disorder due to benzalkonium chloride (BAC) as a preservative has been reported [2].

**Figure 3:**
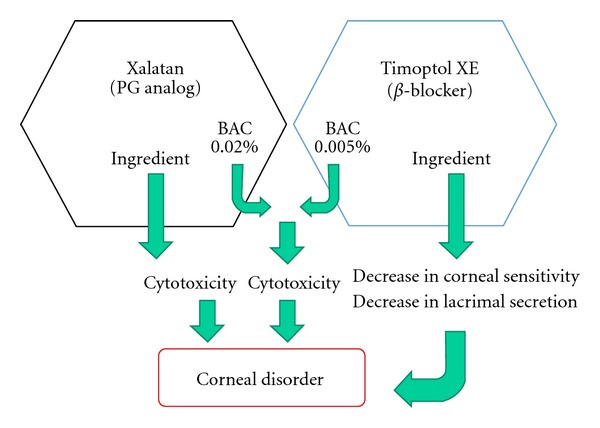
Although corneal disorder can be induced by the cytotoxicity of the main component and BAC in Xalatan eye drops, the cytotoxicity of BAC in Timoptol XE eye drops, or decreases in corneal sensitivity and lacrimal secretion due to the main component of the latter, no corneal disorder developed in this patient.

 In this patient, there was also a possibility that corneal disorder was caused by the corneal cytotoxicity of Xalatan eye drops and Timoptol XE eye drops and decreased sensitivity and aggravation of the lacrimal fluid environment due to the latter. However, according to the previous physician, neither eye pain nor corneal epithelial disorder was observed even during long-term combination therapy with the two drugs before a change from Xalatan to Latanoprost eye drops (Figure 3). After this change, corneal disorder developed only in the eye treated with Latanoprost eye drops in combination with Timoptol XE eye drops, and not in the eye treated with Latanoprost eye drops alone. In addition, after resumption of Latanoprost eye drops, corneal disorder occurred again in the left eye, causing eye pain. Before improvement in the corneal disorder, combination therapy with Xalatan eye drops and Timoptol XE eye drops was performed, but no corneal disorder was observed. This clinical course cannot exclude the possibility of the involvement of Timoptol XE eye drops or Latanoprost eye drops as a generic drug in the development of corneal disorder.

Generic drugs are pharmaceutical drugs that contain the same main component as that of brand-name drugs and are marketed by pharmaceutical companies other than those for brand-name drugs after the completion of the reexamination of brand-name drugs and expiration of patents on their components. At present, various generic drugs are also commercially available in the ophthalmology field. When eye drops are developed, various additives are used in addition to the main component to allow management at higher temperatures and increase the expiry date. As additives, surfactants, thickening agents, isotonizing agents, preservatives, buffers, and stabilizers are used. However, only the main component should be the same between brand-name and generic drugs. Clinical trials on the safety of generic drugs similar to those on the safety of brand-name drugs are not necessary and have not been performed.

Table 1 shows differences in components between Xalatan eye drops as a brand-name drug and Latanoprost eye drops (Kaken Pharmaceutical Co., Ltd.). The concentration of Latanoprost as the main component is the same (0.005%) between the two drugs. As a preservative, benzalkonium chloride is used in both drugs, but information on its concentration in Latanoprost eye drops is not open to the public. Stearic acid polyesters as a surfactant are contained in Latanoprost eye drops but not in Xalatan eye drops.

**Figure 4:**
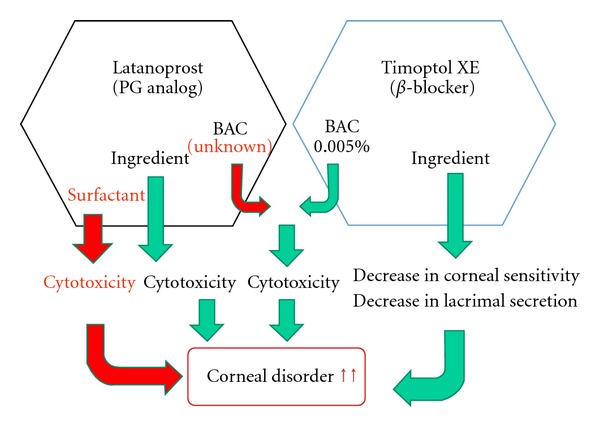
Mechanism of the development of corneal epithelial disorder suggested by the clinical history of this patient. The cytotoxicity of the surfactant may have added to the cytotoxicity of the main component and BAC in Lataronrost eye drops similar to that of Xalatan eye drops. Its use in combination with Timoptol XE eye drops, which decreases corneal sensitivity and lacrimal secretion, may have induced corneal disorder.

Corneal disorder due to surfactants contained in cleaning preparations for contact lenses has been known [6]. Recent studies have shown corneal disorder due to additives contained in eye drops [7]. In this patient, a change to Latanoprost eye drops may have enhanced the corneal cytotoxicity of polyoxyl 40 stearate as a surfactant, resulting in the development of eye pain and corneal disorder (Figure 4). After a change to Xalatan eye drops and Timoptol eye drops again, no corneal disorder was observed. These findings suggest the involvement of Latanoprost eye drops in the development of corneal disorder. In the future, more detailed studies are necessary.

We encountered a patient who developed corneal disorder after a change from Xalatan eye drops as a brand-name drug to Laparoprost eye drops (Kaken Pharmaceutical Co., Ltd.) as its generic drug. Eye drops contain surfactants and additives in addition to the main component. Generic and brand-name drugs are the same in the main component but sometimes differ in other components. When generic drugs are prescribed, differences between the brand-name and generic drugs should be understood. Physicians who prescribe drugs should give consideration to not only the main component, but also preservatives and additives.

## Figures and Tables

**Table 1:** Differences between Latanoprost eye drops (Kaken pharmaceutical Co., Ltd.) and Xalatan eye drops.

	Latanoprost eye drops	Xalatan eye drops
Additives	Benzalkonium chloride additive (concentration not open to the public)	Benzalkonium chloride additive (0.02%)
	Polyethylene glycol monostearate	Isotonizing agent
	Polyoxyl 40 stearate	Dibasic sodium phosphate hydrate
	Isotonizing agent	Sodium dihydrogen phosphate anhydrous
	Dibasic sodium phosphate hydrate	
	Sodium dihydrogen phosphate anhydrous	

Osmotic pressure ratio	0.9–1.0	1.0

Storage	Room temperature storage/protection from light	2–8°C/protection from light
